# Single site laparoscopic right hemicolectomy: an oncological feasible option

**DOI:** 10.1186/1477-7819-8-79

**Published:** 2010-09-08

**Authors:** Yon Kuei Lim, Kheng Hong Ng, Kong Weng Eu

**Affiliations:** 1 Department of Colorectal Surgery, Singapore General Hospital, Outram Road, Singapore

## Abstract

**Introduction:**

We present the first 7 cases of single site right hemicolectomy in Asia using the new Single Site Laparoscopy (SSL) access system from Ethicon Endo-surgery.

**Methods:**

Right hemicolectomy was performed using the new Single Site Laparoscopy (SSL) access system. Patient demographics, operative time, histology and post operative recovery and complications were collected and analysed.

**Results:**

The median operative time was 90 mins (range 60 - 150 mins) and a median wound size of 2.5 cm (range 2 to 4.5 cm). The median number of lymph nodes harvested was 24 (range 20 to 34 lymph nodes). The median length of proximal margin was 70 mm (range 30 to 145 mm) and that of distal margin was 50 mm (35 to 120 mm). All patients had a median hospital stay of 7 days (range 5 to 11) and there were no significant perioperative complications except for 1 patient who had a minor myocardial event.

**Conclusion:**

Right hemicolectomy using SSL access system is feasible and safe for oncologic surgery.

## Introduction

Since the advent of laparoscopy, there have been advances and interest in minimizing the size and number of access sites[1]. With the development of technical skills, more surgeons are trained and are comfortable doing laparoscopic colectomies, the next progression would be to make surgery less invasive without compromising safety. Minimally invasive colectomies have evolved with hand-assisted laparoscopic, conventional laparoscopic and possibly Natural Orifice Transluminal Endoscopic Surgery (NOTES) in the future. Single site laparoscopic surgery lies between conventional laparoscopic and NOTES, and aims to combine the advantages of both approaches [2]. Here, we used the umbilicus, an embryologically natural orifice as the sole access in performing right hemicolectomy for our series of patients, which is the first in Asia using the latest SSL access system.

## Methods

**Figure 1:**
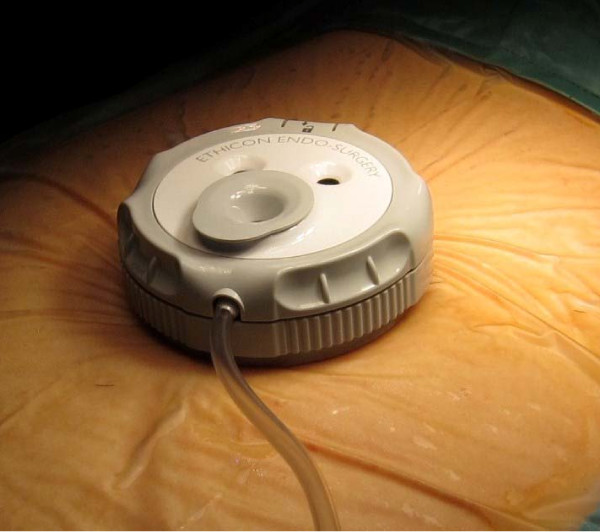
**Seal Cap**.

**Figure 2:**
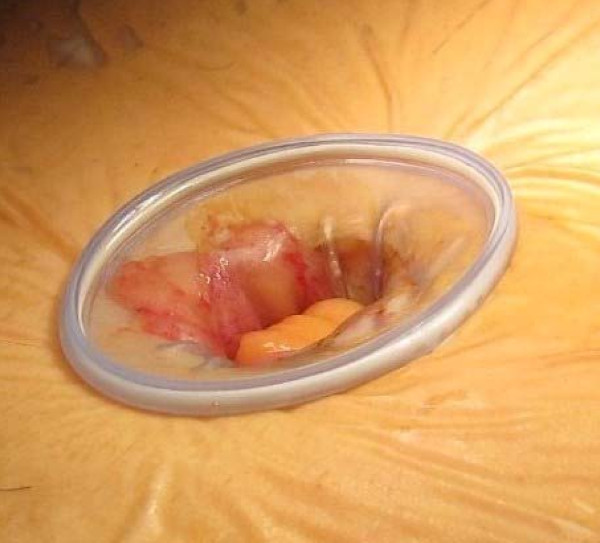
**Fixed Length retractor**.

From April till June 2010, we performed right hemicolectomy on 7 patients using the new SSL access system. In our centre, we performed approximately 220 laparoscopic colectomies per annum. The inclusion criteria were that of a preoperative diagnosis of colonic neoplasm, as well as feasibility for single site approach assessed after a diagnostic laparoscopy at the start of surgery. The SSL access system (ETHICON ENDO- SURGERY Inc, Cincinnati, OH, USA) consist of 2 main components: a seal cap with accessories, and a fixed length retractor. The seal cap consist of (2) 5 mm seals and (1) 5 to 15 mm seal within the inner seal housing (figure 1). A reducer cap is preattached to the 15 mm seal to accommodate use with a 5 mm instrument. A stopcock valve is compatible with the standard luer lock fittings and provides attachment for gas insufflations and desufflation. The fixed length retractor consists of 2 flexible rings interconnected by means of a silicon sleeve (figure 2). Here we used the 2 cm diameter retractor, which allows for an abdominal wall thickness of up to 4 cm. No financial conflict nor support was received from the device manufacturer.

**Figure 3:**
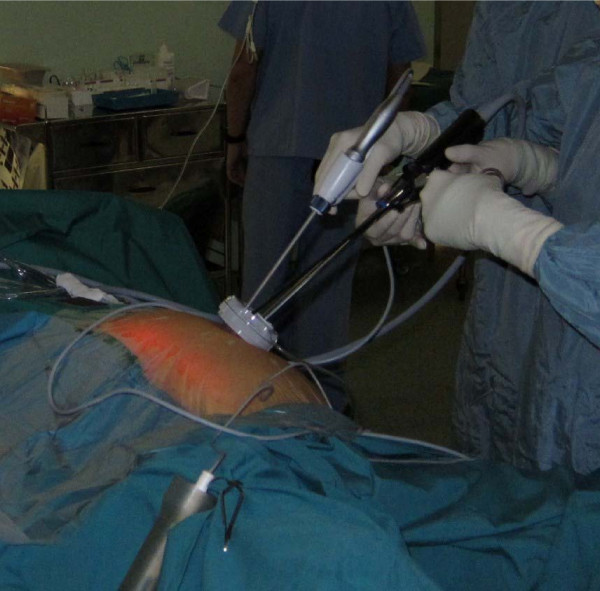
**External view of instruments**.

We approached with an umbilical incision and a Hassan port was inserted with a camera to evaluate feasibility of a laparoscopic resection. If deemed suitable, the umbilical incision was extended till between 1.5 to 2 cm in size and the SSL access system used. The operating table was rotated to obtain a left side down position to allow gravity to retract the small bowel away. Mobilisation of the right colon was performed using the harmonic scalpel and straight laparoscopic graspers (figure 3). We performed a medial to lateral approach. The ileocolic and right colic vessels were divided intracorporeally where possible using a laparoscopic vascular stapler, after the duodenum is identified and protected. In the medial approach, the dissection is carried out toward the hepatic flexure, followed by the lateral mobilisation. Finally the transverse colon is mobilized and the hepatic flexure taken down. The entire specimen was then exteriorized and an extracorporeal anastomosis performed.

## Results

**Figure 4:**
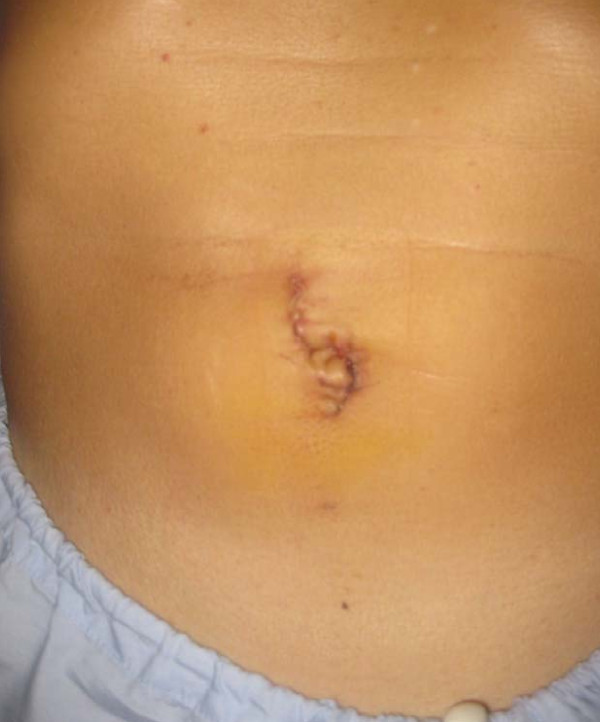
**Post-operative wound**.

There were 4 males and 3 females with a median age of 62(range 63 -79) years who underwent right hemicolectomy with the SSL access system. They had an ASA score of 2 or less. The median Body Mass Index (BMI) was 22 (range 20.1 -30). The indication for surgery for one patient was for a large flat tubular adenoma measuring 2 by 5 cm in size at the hepatic flexure. The rest had adenocarcinoma of the right colon, with a median tumour length of 3.5 cm and width of 2.5 cm. The median operative time was 90 mins (range 60 - 150 mins), slight blood loss(less than 20 mls) and a median wound size of 2 cm (range 2 to 2.5 cm) (figure 4). Histology revealed adenocarcinoma in 6 of the 7 specimens and 1 was that of tubular adenoma with low grade dysplasia. The median number of lymph nodes harvested was 24 (range 20 to 34 lymph nodes). The median length of proximal margin was 70 mm (range 30 to 145 mm) and that of distal margin was 50 mm (35 to 120 mm).

Postoperatively, the patients had a median pain score of 2(out of 10) on the day of the operation, and a score of 2 on the 1^st ^postoperative day. All had no significant pain after the 2^nd ^post-operative day. All patients had a median hospital stay of 7 days (range 5 to 11 days) and there were no significant perioperative complications, other than 1 patient who had a minor myocardial infarct. Specifically, none of the patients had surgical site infection. All patients stayed beyond commencement of bowel function for social reasons.

## Discussion

Single port access right hemicolectomy has been shown to be feasible for oncologic surgery [3]. It may have advantage over conventional laparoscopic surgery in terms of reduced pain, lower cost, faster recovery and cosmesis [4]. There have been many different types of single port access systems, each trying to improve on previous models. Here, we evaluate the latest refinement, which is the SSL access system.

There are several advantages using the new SSL access system. The low profile seal cap enables us to use a wide range of instruments. The integrated system also eliminates the needs for trocars which might interfere with the manipulation of instruments in the abdominal cavity. Furthermore, 360 degrees seal cap rotation allows quick reorientation of instruments throughout the surgery without requiring instrument exchanges. At the end of the procedure, the retractor of the SSL access system also serves as a wound protector during specimen retrieval.

Here, we also demonstrate that single port right hemicolectomy is feasible with a reasonable operative time (median 90 mins), and no significant perioperative complications. Based on the histopathological report, there were adequate lymph nodes (median 24 lymph nodes) and resection margins (median proximal 70 mm, distal 50 mm) from the resected specimens. Our results are comparable to other case reports and case series in the literature [5-7], which have reported operative time ranging from 115 to 255 mins.

In terms of patient preference, single port appendicectomy has been shown to be the most favoured method over open, conventional laparoscopic and NOTES, extrapolating, perhaps this may be the approach to invest in [8] for laparoscopic colectomies in the future.

## Conclusion

Right hemicolectomy using SSL access system is feasible and safe for oncologic surgery. It has encouraging results in terms of operating time and postoperative pain score. The favourable results thus far suggest this may be the direction for the future of minimally invasive colorectal surgery.

## Competing interests

The authors declare that they have no competing interests.

## Authors' contributions

LYK wrote the draft, did the data collection and analysed the results, performed the statistical analysis, and finally wrote the final manuscript. NKH conceived the idea and intellectual content as well as contributed in surgery and contributed ideas to the manuscript. He participated in the design of the study and coordination and helped to draft the manuscript. EKW interpreted the data, assisted in coordination and revised the final manuscript for intellectual content. All authors read and approved the final manuscript.
